# Educational Level Mediates the Relationship Between Knowledge and Preventive Practices in Multidrug-Resistant Tuberculosis Patients

**DOI:** 10.3390/epidemiologia6040075

**Published:** 2025-11-06

**Authors:** Raquel Clemencia Guardia Zuñiga, Blanca Victoria Abad de Vite, Amariles Azañero Suarez, Danicsa Karina Espino Carrasco, Esther García Santos, Idalia Eufemia Lajo Aquise, Irma Cachay Sánchez

**Affiliations:** 1 Escuela de Enfermería, Facultad de Ciencias de la Salud, Universidad César Vallejo, Piura 20000, Peru; rcguardiag@ucvvirtual.edu.pe (R.C.G.Z.); aazaneros@ucvvirtual.edu.pe (A.A.S.); 2 Escuela de Enfermería, Facultad de Ciencias de la Salud, Universidad Nacional de Piura, Piura 20000, Peru; babadq@unp.edu.pe (B.V.A.d.V.); egarciasa@unp.edu.pe (E.G.S.); ilajoa@unp.edu.pe (I.E.L.A.); icachaisa@unp.edu.pe (I.C.S.)

**Keywords:** multidrug-resistant tuberculosis, knowledge, preventive practices, educational level, health care settings, patient education, treatment adherence, mediation analysis, public health, disease prevention

## Abstract

Background: Multidrug-resistant tuberculosis (MDR-TB) prevention presents significant challenges in Peruvian healthcare settings, with substantial gaps in knowledge implementation and preventive practices. However, little is known about how patients’ sociodemographic factors influence the translation of knowledge into preventive practices. Methods: This cross-sectional study examined the associations between knowledge, preventive practices, and potential mediating roles of occupation, educational level, and sex among MDR-TB patients. We surveyed 280 patients from twelve health centers in the Piura-Castilla Network, Peru, recruited from urban (38.55%), marginal urban (32.06%), and rural (29.39%) areas through nonprobability convenience sampling. Participants represented diverse occupational backgrounds, including housewives (19.85%), workers (20.99%), and unemployed individuals (23.28%). Results: Measurement instruments were validated through confirmatory factor analysis, demonstrating adequate reliability (McDonald’s ω > 0.80) and discriminant validity (HTMT < 0.85). Path analysis using structural equation modeling assessed direct and indirect relationships. Knowledge showed a significant direct association with preventive practices (β = 0.194, *p* < 0.001). Among the three mediating variables examined, only educational level demonstrated a significant indirect effect (β = 0.073, *p* < 0.001), while occupation (β = −0.010, *p* = 0.490) and sex (β = −0.035, *p* = 0.150) showed no significant indirect associations. The model explained 29.7% of the variance in preventive practices. Conclusions: Educational level appears to facilitate the translation of knowledge into preventive practices among MDR-TB patients, though the cross-sectional design precludes causal or directional inferences. Healthcare institutions should develop tailored educational interventions according to patients’ educational backgrounds, including literacy-sensitive materials, simplified visual aids, and personalized counseling sessions to enhance MDR-TB prevention effectiveness in clinical settings.

## 1. Introduction

Multidrug-resistant tuberculosis (MDR-TB) represents a critical challenge for health systems internationally and is characterized by significant deficiencies in knowledge and preventive practices. Globally, the World Health Organization reported approximately 410,000 incident cases of rifampicin-resistant tuberculosis (RR-TB) in 2022, with MDR-TB accounting for the majority of these cases and treatment success rates remaining suboptimal at 63% for MDR/RR-TB cohorts enrolled in 2020 [1]. In the Latin American context, Peru ranks among the 30 high MDR-TB burden countries globally, with an estimated 3200 incident MDR/RR-TB cases in 2022 and a treatment success rate of 69% for cases enrolled in 2020 [1]. These concerning epidemiological indicators underscore the urgent need to understand factors that influence preventive behaviors at the patient level. While existing research has documented knowledge deficiencies among healthcare workers—with less than 50% demonstrating adequate MDR-TB knowledge in Lesotho and only 39.5% in Ethiopia [2,3]—far less attention has been directed toward understanding knowledge and preventive practices among patients themselves, who constitute the primary population at risk for disease transmission and treatment failure. This situation is aggravated by the inadequate implementation of infection control measures in settings with limited resources, where a lack of experience, funding and resources hinders the effective application of the WHO guidelines [4,5]. Studies have revealed that economic barriers have a significant effect on the management and prevention of MDR-TB, with low socioeconomic status being a factor associated with increased rates of drug-resistant TB [6,7].

The current scientific literature presents significant gaps in the understanding of the associations between knowledge and preventive practices in patients with MDR-TB, particularly within the Peruvian context. Although some studies have shown that a lack of knowledge constitutes a risk factor for treatment abandonment [8,9], there is a critical need to investigate how sociodemographic variables are associated with preventive practices, specifically among Peruvian patient populations. However, little is known about how patients’ sociodemographic characteristics—particularly educational level, occupation, and sex—may facilitate or hinder the translation of disease knowledge into actual preventive behaviors. This gap becomes more evident in Peru when considering that only 19.6% of health workers report good practices in prevention and control in similar resource-limited settings [2], whereas studies in various international contexts highlight the importance of factors such as social support and patient education [10,11]. However, the potential mediating role of sociodemographic factors in the knowledge-practice association remains largely unexplored in the Peruvian healthcare context, where unique cultural, educational, and socioeconomic characteristics may shape these relationships differently than in other populations.

The theoretical framework underlying this research draws upon the Health Belief Model and Social Cognitive Theory, which posit that health behaviors result from the interaction between individual knowledge, perceived self-efficacy, and sociodemographic factors that shape access to and processing of health information [12,13]. Knowledge about MDR-TB is operationally defined as patients’ comprehensive understanding of disease transmission mechanisms, treatment protocols, infection control measures, and the critical importance of adherence to prevent further resistance development [2]. Preventive practices encompass specific behaviors aimed at reducing MDR-TB transmission, including proper use of protective measures, adherence to treatment regimens, and implementation of household infection control strategies [3]. The selection of mediating variables is theoretically grounded: educational level influences health literacy and information processing capacity [14]; occupation determines exposure to health information and resources for implementing preventive behaviors [15]; and sex affects health-seeking behaviors and social roles that influence preventive practice adoption [16]. These three variables were prioritized over other sociodemographic factors (such as income, ethnicity, or general health literacy) based on theoretical frameworks suggesting they represent distinct pathways through which knowledge may be translated into behavior: educational level affects cognitive processing and comprehension of health information, occupation shapes daily routines and resource access that enable practice implementation, and sex influences social roles and healthcare engagement patterns. Furthermore, these variables are consistently collected in tuberculosis surveillance systems, enhancing the practical applicability of our findings. We conceptualize these variables as mediators rather than moderators or background characteristics because theory suggests they represent mechanisms or pathways through which knowledge operates on behavior, rather than conditions that strengthen or weaken the knowledge-practice relationship (moderation) or simple demographic descriptors (background variables). This theoretical foundation suggests that while knowledge provides the cognitive basis for preventive behaviors, sociodemographic factors serve as pathways through which knowledge is translated into actual preventive practices, creating distinct mediating mechanisms that warrant empirical investigation in the MDR-TB context.

The main constructs of this research include knowledge about multidrug-resistant tuberculosis (MDR-TB) and preventive practices, which specifically analyze how these factors are associated with potential mediating variables such as occupation, educational level and sex of the patients. The evidence suggests that these sociodemographic variables may be associated with treatment outcomes and preventive practices [17].

In the Peruvian context, the problem presents specific and complex characteristics. Studies have shown that therapeutic abandonment is significantly associated with a lack of knowledge about the disease and the absence of social support [9,18]. The geographical distribution of MDR-TB in Peru shows specific patterns, with a higher prevalence in regions such as the central jungle [19]. Studies with health personnel in Lima have revealed significant gaps in knowledge and attitudes toward tuberculosis [20], whereas recent research has identified critical factors associated with knowledge and practices related to tuberculosis in the Peruvian population [21].

The general objective of this research is to examine the potential mediating role of occupation, educational level and sex in the association between knowledge and preventive practices for multidrug-resistant tuberculosis in treated patients in health establishments in Piura. The specific objectives include analyzing the distribution of sociodemographic variables, determining the coefficients of determination of the mediation model and establishing the direct association between knowledge and preventive practices. This study represents a significant advance in addressing the complex interaction between sociodemographic variables and their potential associations with preventive practices, especially considering the local context and the specific characteristics of the population of Piura.

The theoretical justification of the study lies in its contribution to the understanding of potential mediation mechanisms between sociodemographic variables and their associations with the relationship between knowledge and preventive practices in MDR-TB. From a practical perspective, the results will allow the development of more effective and personalized interventions according to the characteristics of the patients, addressing the recommendations of previous studies on the need to strengthen infection control measures [22] and improve the management of household contacts [23]. These findings will be particularly relevant for optimizing prevention and control strategies in health facilities in Piura, helping to reduce the burden of the disease and improve treatment results [24].

## 2. Literature Review

### 2.1. Knowledge and Multidrug-Resistant Tuberculosis

MDR-TB represents a particularly challenging variant of tuberculosis characterized by resistance to the two most potent antituberculosis drugs: isoniazid (INH) and rifampin (RIF). This condition can evolve into an even more severe form, known as extensively resistant tuberculosis (XDR-TB), which presents additional resistance to any fluoroquinolone and at least one of the three second-line injectable drugs [25,26].

Knowledge about MDR-TB is defined as a comprehensive understanding of the fundamental aspects of the disease, including its etiology, transmission, prevention and treatment. This conceptual framework encompasses patients’ awareness of drug resistance mechanisms, treatment duration requirements, infection control measures, and the critical importance of treatment adherence to prevent further resistance development. In empirical research, knowledge has been operationalized and measured through various validated instruments. Studies with healthcare workers have employed structured questionnaires assessing factual understanding of transmission routes, infection control protocols, and treatment regimens [2,3], while patient-focused research has adapted these instruments to evaluate lay understanding using simplified terminology and response formats appropriate for diverse educational backgrounds [27]. Measurement typically encompasses multiple dimensions: awareness of disease causation and transmission pathways, understanding of treatment duration and medication adherence requirements, recognition of infection control measures for household settings, and comprehension of drug resistance mechanisms. Studies have shown that the level of knowledge is influenced by various factors, such as educational level, training in infection prevention, and personal experience with the disease [2]. Recent systematic reviews have identified significant knowledge gaps among both healthcare workers and patients, with knowledge scores varying substantially across different populations and healthcare settings [27,28]. Importantly, patient knowledge levels have been consistently documented as lower than those of healthcare providers, with studies reporting that fewer than 40% of MDR-TB patients demonstrate adequate understanding of their disease condition and preventive measures [27]. In the Peruvian context, this understanding becomes particularly crucial given the country’s significant MDR-TB burden. According to the World Health Organization’s Global Tuberculosis Report 2023, Peru ranks among the 30 high MDR-TB burden countries globally, with an estimated 3200 incident MDR/RR-TB cases in 2022 and a treatment success rate of 69% for MDR/RR-TB cases enrolled in 2020 [1], highlighting the urgent need for effective knowledge-based interventions to improve preventive practices among affected populations.

### 2.2. Preventive Practices in MDR-TB Management

Preventive practices comprise a set of specific actions aimed at reducing the transmission and spread of MDR-TB. These include the proper use of personal protective equipment, adherence to treatment protocols, and the implementation of infection control measures [3]. Contemporary research emphasizes the multifaceted nature of preventive practices, incorporating both individual-level behaviors and community-based interventions [29,30]. Among patient populations specifically, preventive practices encompass medication adherence, covering the mouth when coughing, maintaining adequate ventilation in living spaces, attending scheduled medical appointments, and implementing household infection control strategies to protect family members [3,11]. Research has shown that barriers to adherence to these preventive practices include the feeling of invulnerability among health care personnel, low provider-to-patient ratios, and limited availability of protective equipment [31]. Recent evidence from low- and middle-income countries indicates that systematic implementation of infection prevention and control measures significantly reduces nosocomial transmission rates when combined with adequate training and resource allocation [32].

The relationship between knowledge and preventive practices is based on various studies that suggest a positive association between both variables. Research has shown that inadequate knowledge about MDR-TB can result in poor preventive practices and increase the risk of transmission [33]. This relationship is influenced by environmental and socioeconomic factors, which can affect both the acquisition of knowledge and the implementation of effective preventive practices [34]. Recent longitudinal studies have demonstrated that structured educational interventions significantly improve both knowledge retention and preventive behavior adoption among MDR-TB patients [11,35]. While previous descriptive research has documented correlations between knowledge and practices, examining potential mediating pathways offers theoretical advancement by identifying specific mechanisms through which knowledge may translate into behavior, thereby informing more precisely targeted interventions that address intermediate factors rather than solely focusing on knowledge transfer.

**H1.**  *Knowledge is positively associated with preventive practices among multidrug-resistant tuberculosis patients receiving treatment.*

### 2.3. Sociodemographic Mediating Factors

The potential mediating role of sociodemographic variables in the association between knowledge and preventive practices for multidrug-resistant tuberculosis (MDR-TB) has been the subject of several studies that demonstrate its importance in the effectiveness of health interventions. Research in this field has identified specific patterns of potential mediation that deserve detailed analysis. Exploring mediation rather than simple description or moderation provides novel theoretical insight by revealing the pathways and mechanisms through which knowledge operates on behavior, moving beyond documenting that relationships exist to understanding how they function through intermediate variables.

### 2.4. Educational Level as a Mediator

Educational level emerges as a crucial potential mediator in the effectiveness of the transmission and application of knowledge about MDR-TB. Research specific to patient populations has demonstrated that individuals with higher educational attainment exhibit enhanced capacity to comprehend medical information, retain complex health instructions, and implement multistep preventive behaviors [14]. Among MDR-TB patients specifically, educational level has been associated with better treatment adherence and more consistent implementation of household infection control measures [9,18]. This finding suggests that educational level, through its influence on health literacy and information processing capacity, may enhance the translation of disease knowledge into preventive practices. Contemporary research confirms that health literacy, closely linked to educational attainment, serves as a critical pathway for translating medical knowledge into actionable health behaviors [36]. Studies examining tuberculosis patients rather than healthcare workers or students have documented that those with secondary or higher education demonstrate significantly better adherence to medication regimens and protective measures compared to patients with primary education or no formal schooling [20]. The mediating mechanism operates through enhanced comprehension of written materials, improved communication with healthcare providers, and greater capacity to problem-solve barriers to practice implementation [37,38].

### 2.5. Occupation as a Mediating Variable

As a potential mediating variable, occupation may influence the association between knowledge and preventive practices through multiple pathways. Among patient populations, occupational status determines several factors relevant to preventive behavior implementation: daily routines and schedules that affect healthcare appointment attendance, financial resources available for transportation and nutrition, physical demands that may exacerbate disease symptoms, and social networks that provide support or stigma [15]. In the context of health personnel, workers with higher levels of education and specific training in infection prevention exhibit better levels of knowledge and practices related to MDR-TB [2]. This relationship extends to the nonhealth field, where workers with higher educational levels demonstrate greater understanding and application of preventive measures [39]. For MDR-TB patients specifically, employment status has been associated with treatment outcomes, with unemployed patients facing greater challenges in maintaining consistent preventive practices due to competing survival priorities and reduced access to resources [9,18]. Recent studies have shown that occupation significantly influences the awareness and application of preventive measures against tuberculosis, with healthcare workers demonstrating superior knowledge and practice implementation compared to other occupational groups [40,41]. However, the mechanisms through which occupation operates as a mediator in patient populations require further investigation, particularly considering that unemployment and informal employment predominate among MDR-TB patients in resource-limited settings.

### 2.6. Sex-Based Differences in Health Behaviors

Regarding sex as a potential mediating variable, studies have revealed distinctive patterns in the association between knowledge and preventive practices. Among patient populations, sex influences health behaviors through socially constructed roles, differential healthcare access patterns, and varying responsibilities for household management and childcare that affect the capacity to implement preventive measures [16]. Recent research has shown that female high school students tend to score higher in preventive knowledge and practices than their male counterparts do [42]. However, this gender difference is not universal; some studies with medical students have not reported significant differences between genders in terms of knowledge and practices [43]. In tuberculosis patient populations specifically, studies have yielded mixed findings, with some documenting that female patients demonstrate better medication adherence while others report no significant sex-based differences in preventive behaviors [21]. Meta-analytic evidence suggests that sex-based differences in health behavior adoption are context-dependent and may be influenced by cultural factors and healthcare system characteristics [44]. The potential mediating role of sex in the knowledge-practice relationship among MDR-TB patients warrants investigation within specific cultural contexts, as gender norms governing health responsibilities and healthcare engagement vary substantially across populations.

The evidence presented here supports the following specific hypotheses:

**H2.**  *Occupation is associated with the relationship between knowledge and preventive practices for multidrug-resistant tuberculosis in patients receiving treatment at a health center through potential indirect pathways.*

**H3.**  *Educational level is associated with the relationship between knowledge and preventive practices for multidrug-resistant tuberculosis in patients receiving treatment in a health center through potential indirect pathways.*

**H4.**  *Sex is associated with the relationship between knowledge and preventive practices for multidrug-resistant tuberculosis in patients receiving treatment in health centers through potential indirect pathways.*

**Figure 1:**
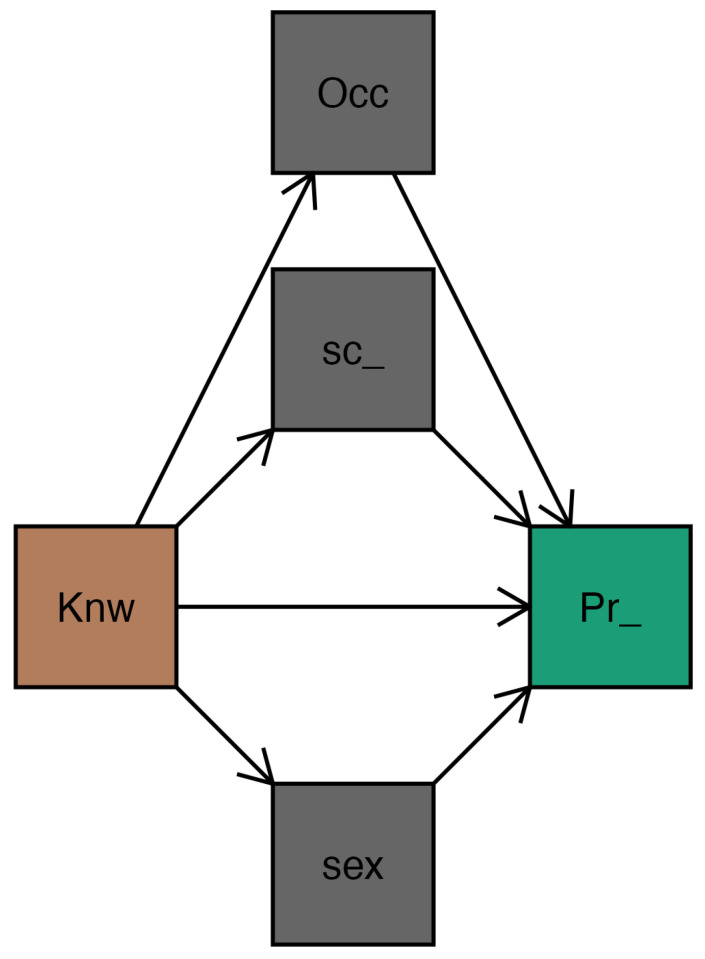
Conceptual path plot. Note. Knw = Knowledge; Pr_ = Preventive_practices; sex = sex; Sc_ = school_level; Occ = Occupation.

Figure 1 shows the proposed mediation model where the direct and indirect hypotheses are supported.

## 3. Materials and Methods

### 3.1. Study Design

This research employed a cross-sectional, quantitative design to examine associations between knowledge, preventive practices, and sociodemographic factors among MDR-TB patients. Understanding how knowledge relates to preventive practices in multidrug-resistant tuberculosis requires rigorous analysis of the factors associated with its adoption. To examine how knowledge may be associated with preventive practices, an empirical evaluation was implemented, following the methodological recommendations established in the literature for research on health behaviors in patient populations [45]. The analytical framework utilized path analysis to evaluate direct associations and potential indirect pathways through which sociodemographic variables may be linked to the knowledge-practice relationship. Path coefficients were calculated to quantify the strength and direction of associations, where direct effects represent the unmediated relationship between knowledge and preventive practices, while indirect pathways capture associations that operate through sociodemographic variables (occupation, educational level, and sex). This approach allows for the simultaneous estimation of multiple pathways and provides standardized coefficients that facilitate the interpretation of effect sizes across different variables, ensuring a comprehensive understanding of the complex associations between knowledge, sociodemographic factors, and preventive behaviors in the MDR-TB context. We emphasize that the cross-sectional design precludes causal inference; observed associations reflect correlational patterns rather than directional or causal relationships.

### 3.2. Participants

A total of 280 patients diagnosed with MDR-TB participated in this study, representing approximately 8.2% of all MDR-TB patients receiving treatment in the Piura region during 2022–2023, based on regional tuberculosis program records indicating approximately 3200 active MDR/RR-TB cases [28]. Sample size adequacy was evaluated using G*Power 3.1, which confirmed sufficient statistical power (1-β = 0.89) for detecting medium effect sizes (f^2^ = 0.15) in multiple regression analyses with three predictors at α = 0.05. This sample size exceeds the recommended minimum of 200 participants for stable parameter estimation in path analysis models [46], supporting the robustness of our statistical analyses.

Participants were recruited from twelve health centers within the Piura-Castilla Network, specifically located in the districts of Piura, Veintiséis de Octubre, and Castilla. Patient selection was conducted through the Estrategia Sanitaria Nacional de Prevención y Control de la Tuberculosis (ESNPCT) coordinators at each health center, who identified eligible patients during their scheduled treatment supervision visits. This recruitment strategy leveraged the existing directly observed treatment (DOT) framework, where MDR-TB patients attend regular appointments for medication administration and monitoring.

### 3.3. Inclusion and Exclusion Criteria

Inclusion criteria comprised the following: (1) confirmed MDR-TB diagnosis through laboratory testing showing resistance to at least rifampin and isoniazid; (2) age ≥ 18 years; (3) active treatment status registered in the national tuberculosis control program; (4) voluntary informed consent; and (5) cognitive capacity to understand questionnaire items. Exclusion criteria included: (1) patients with severe psychiatric conditions impairing comprehension; (2) individuals declining participation after informed consent procedures; (3) patients with incomplete treatment records; and (4) inability to communicate in Spanish.

The study examined three sociodemographic variables—occupation, educational level, and sex—as potential statistical mediators based on theoretical frameworks suggesting their relevance in health behavior patterns [2,37]. We acknowledge that these variables are more appropriately conceptualized as antecedent factors that may influence both knowledge acquisition and practice implementation, rather than as variables that could be modified by knowledge or practices. Educational level and sex represent stable demographic characteristics established prior to MDR-TB diagnosis, while occupation reflects socioeconomic position that shapes access to health resources. Our analytical framework examines statistical pathways through which these pre-existing characteristics may be associated with the knowledge-practice relationship, without implying temporal precedence or causal mechanisms. While other sociodemographic factors such as age, place of residence, and ethnicity were collected for sample characterization, they were not included in the mediation analysis due to (1) the exploratory nature of this initial study requiring a focused analytical approach, (2) sample size limitations for complex multi-mediator models, and (3) the theoretical emphasis on educational and occupational pathways in knowledge-to-practice translation identified in previous literature. Future studies should examine these additional variables as potential correlates.

Additionally, all participants were asked about their perceived understanding of how they developed MDR-TB, including whether they believed inadequate preventive practices contributed to their condition. This self-reflection component provided valuable context for interpreting knowledge-practice relationships among individuals who had experienced treatment failure or primary resistance. Following established methodological frameworks for health behavior research in vulnerable populations, nonprobability convenience sampling was implemented [46].

### 3.4. Instruments

#### 3.4.1. Operational Definition

For this study, MDR-TB patients were defined as individuals with laboratory-confirmed tuberculosis showing resistance to at least rifampin and isoniazid, the two most potent first-line anti-tuberculosis drugs, as determined by drug susceptibility testing and registered in the national tuberculosis control program.

#### 3.4.2. Instrument Development and Validation

The research instruments were adapted from previously validated scales focusing on knowledge and practices regarding MDR-TB, originally developed for healthcare workers [1,2]. Given that the original instruments targeted healthcare personnel, substantial adaptations were made to ensure appropriateness for the general patient population. Language was simplified to accommodate varying educational levels, medical terminology was replaced with lay terms, and content was modified to reflect patient experiences rather than healthcare provider perspectives. For example, items about “implementing infection control protocols” were adapted to “following protective measures at home.”

### 3.5. Questionnaire Structure and Administration

The questionnaire began with a sociodemographic section gathering basic participant information through 7 items, including age, sex, marital status, educational level, place of residence, and occupation. The knowledge assessment component comprised 12 items evaluating understanding of disease transmission, treatment protocols, and prevention measures, with responses scored on a 5-point Likert scale (1 = strongly disagree to 5 = strongly agree). Sample items included “I understand how tuberculosis spreads from person to person” and “I know the importance of completing my entire treatment regimen.” The preventive practices section included 12 items measuring both household hygiene behaviors and health service utilization patterns via a three-point frequency scale (always, sometimes, never). Representative items included “I cover my mouth when coughing” and “I attend all scheduled medical appointments.”

The questionnaire was administered through face-to-face interviews by trained research assistants to ensure comprehension across varying educational levels and to minimize missing data. Each interview lasted approximately 20–25 min and was conducted in a private setting within the health facilities to maintain confidentiality.

Psychometric validation was conducted using confirmatory factor analysis. The knowledge scale demonstrated acceptable average variance extracted (AVE = 0.652), indicating adequate convergent validity, high composite reliability (CR = 0.901), and acceptable discriminant validity as evidenced by heterotrait-monotrait ratio (HTMT = 0.841). Similarly, the preventive practices scale showed good AVE (0.724), excellent CR (0.923), and acceptable HTMT (0.812). Both sections exhibited strong internal consistency during pilot testing, with the knowledge scale showing McDonald’s omega (ω = 0.89) and the practices scale demonstrating robust reliability (ω = 0.91).

### 3.6. Procedure and Data Analysis

#### 3.6.1. Analytical Software and Procedures

Data analysis was conducted using multiple software packages optimized for different analytical phases. Initial data management and descriptive statistics were performed using SPSS 28.0. All subsequent analyses, including confirmatory factor analysis and mediation modeling, were conducted using JASP 0.19.3 [47].

Confirmatory factor analysis with the pilot sample (*n* = 30) was performed to validate the measurement model, which demonstrated satisfactory fit indices (CFI = 0.962, RMSEA = 0.058, TLI = 0.954, SRMR = 0.043). The main mediation analysis employed JASP’s integrated implementation of Hayes’ PROCESS framework [48], specifically Model 4, to examine simple mediation pathways. This approach implements ordinary least squares regression-based path analysis to estimate direct and indirect associations. Bootstrapping with 5000 resamples was used to generate bias-corrected confidence intervals for indirect effects. Path coefficients were calculated via standardized beta weights, with statistical significance assessed through 95% confidence intervals that exclude zero.

We clarify that while structural equation modeling (SEM) concepts informed our analytical framework, the actual statistical implementation utilized regression-based path analysis through JASP’s mediation module rather than full SEM with latent variables. This approach employs ordinary least squares regression to estimate path coefficients and bootstrapping to test indirect effects, providing a robust method for examining associative pathways in mediation frameworks [48]. This method is appropriate for observed variables and offers advantages in computational efficiency and interpretability for straightforward mediation models. In addition, JASP automatically calculates Cohen’s f2 effect sizes for total effects where the magnitude of the observed associations is quantified.

#### 3.6.2. Model Specification and Control Variables

The mediation models controlled for potential confounding variables, including age, marital status, and place of residence, which were entered as covariates in all analyses based on their theoretical relevance identified in previous literature. Model diagnostics included checks for multicollinearity (variance inflation factors < 3.0), residual normality (Shapiro–Wilk tests), and homoscedasticity (Breusch-Pagan tests). The PROCESS macro specifically facilitated the examination of complex mediation pathways while providing bias-corrected bootstrap confidence intervals for indirect effects.

#### 3.6.3. Ethical Considerations

The research protocol received approval from the Ethics Committee of the Instituto de Investigación, Innovación, Ciencia y Tecnología (IIICyT) with approval code 2024-IIICyT-ITCA, and was conducted in accordance with the Declaration of Helsinki principles. All participants provided written informed consent after receiving comprehensive information about the study’s objectives, procedures, potential risks, and their rights as research subjects. Given the sensitive nature of MDR-TB diagnosis, strict confidentiality measures were implemented, including data anonymization and secure storage protocols. Participation was voluntary, and patients were explicitly informed that their treatment would not be affected by their decision to participate or withdraw from the study. Data collection procedures were designed to minimize participant burden while ensuring a comprehensive assessment of study variables

## 4. Results

### 4.1. Sociodemographic Characteristics

Table 1 presents the sociodemographic characteristics of the 280 MDR-TB patients. The mean age was 34.2 ± 12.8 years (range: 18–74 years), with the majority (33.21%) belonging to the 21–30 age group. The sample showed a balanced sex distribution with slightly more males (51.15%) than females (48.85%). Regarding marital status, participants were distributed among single (22.14%), cohabiting (21.75%), widowed (20.23%), married (18.32%), and divorced (17.56%) categories. Educational levels varied considerably, with similar proportions having no formal education (22.14%), primary education (21.37%), and secondary education (20.99%), while smaller proportions had university (19.47%) and technical education (16.03%). Most participants resided in urban areas (38.55%), followed by marginal urban (32.06%) and rural areas (29.39%). Employment status showed that unemployment was the most common condition (23.28%), followed by worker (20.99%), housewife (19.85%), employee (19.08%), and student (16.79%) categories.

### 4.2. Research Model Results

Table 2 presents the results of the summary of the mediation model evaluated in the study. The configuration number of the model defined by [48] was used, specifically model number 4. The adjustment indicators include the Akaike information criterion (AIC), which reached a value of 4080.153, and the Bayesian information criterion (BIC), with a value of 4123.770. Both criteria have a weight of 1000, which indicates a high level of evidence in favor of the selected model compared with alternative models.

The loglikelihood of the model obtained was −2028.076, reflecting the level of fit of the model to the observed data. Similarly, the total number of observations analyzed (*n*) was 280, with 3 degrees of freedom (df), which suggests a parsimonious model in relation to the amount of data available.

These results reflect the adequacy of the proposed mediation model in explaining the relationship between knowledge about multidrug-resistant tuberculosis and preventive practices, considering the influence of the sociodemographic variables included in the analysis.

Table 3 presents the values of the coefficient of determination (R^2^) for the proposed mediation model. The results show that the knowledge variable explains 29.7% of the variability in multidrug-resistant tuberculosis preventive practices, which indicates a moderate level of adjustment. On the other hand, the mediating variables present lower values of R^2^: occupation explains 8.8% of its variability, educational level explains 5.6%, and sex explains 2.8%. These results suggest that although the three mediating variables examined—occupation, educational level, and sex—contribute to explaining the relationship between knowledge and preventive practices, their combined impact remains relatively modest compared to the direct effect of knowledge on preventive practices. Specifically, while educational level demonstrated a significant mediating effect (β = 0.073, *p* < 0.001), occupation (β = −0.010, *p* = 0.490) and sex (β = −0.035, *p* = 0.150) showed no significant mediating effects in this patient population.

Table 4 shows the trajectory coefficients that evaluate the direct relationship between knowledge about multidrug-resistant tuberculosis and preventive practices. The standardized regression coefficient (B = 0.194) indicates a positive and significant association between both variables, with a standard error of 0.052. The value of z obtained (3.701) and the level of significance *p* < 0.001 confirm the statistical robustness of this relationship. Furthermore, the 95% confidence interval (0.091–0.265) supports the accuracy of the estimator, suggesting that knowledge has a positive effect on the adoption of preventive practices.

**Figure 2:**
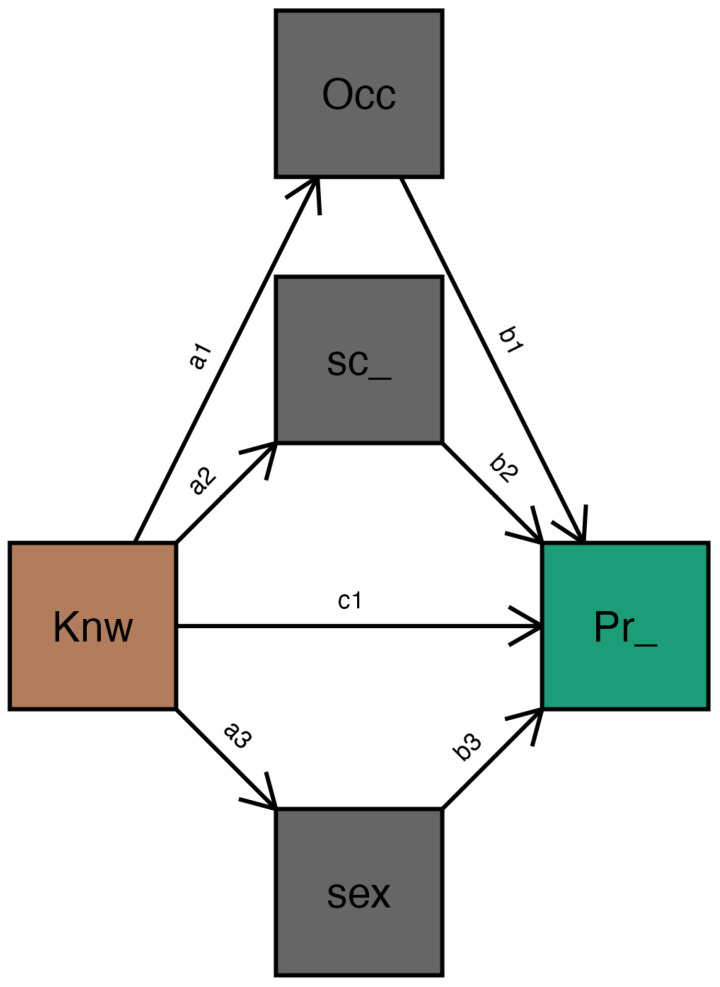
Statistical path plot. Note. Knw = Knowledge; Pr_ = Preventive_practices; sex = sex; Sc_ = school_level; Occ = Occupation.

On the other hand, Figure 2 of the statistical diagram shows this relationship through the coefficient c1, which represents the direct hypothesis proposed in the model. Thus, knowledge (Knw) directly influences preventive practices (Pr_), highlighting the importance of promoting educational strategies to strengthen preventive actions in patients.

Table 5 shows the indirect effects of knowledge on multidrug resistance prevention practices, considering the mediating variables occupation, school level, and sex.

For Hypothesis 2, which states that occupation significantly mediates the relationship between knowledge and preventive practices, the results show a nonsignificant regression coefficient (B = −0.010, *p* = 0.490), with a 95% confidence interval ranging from −0.040 to −0.019. Since the *p* value is greater than the threshold of 0.05, the mediation hypothesis is rejected, which suggests that occupation does not play a significant role in the relationship between knowledge and preventive practices.

In relation to Hypothesis 3, which maintains that the school level significantly mediates this relationship, the results indicate a significant regression coefficient (B = 0.073, *p* < 0.001), with a 95% confidence interval from 0.031 to 0.115. This allows us to accept the hypothesis, confirming that the school level plays a mediating role in the relationship between knowledge and preventive practices.

Finally, for Hypothesis 4, which postulates that sex significantly mediates the influence of knowledge on preventive practices, the results show a nonsignificant regression coefficient (B = −0.035, *p* = 0.150), with a confidence interval of 95% from −0.083 to 0.013. As statistical significance was not reached, this hypothesis is rejected, which indicates that sex does not act as a mediator in the analyzed relationship.

Table 6 shows the total effects of the model, differentiating between the total effect of knowledge on preventive practices and the indirect effects mediated by the variable’s occupation, school level and sex. Cohen’s f^2^ effect size is reported to quantify the magnitude of these effects, where f^2^ values of 0.02, 0.15, and 0.35 represent small, medium, and large effects, respectively.

The total effect of knowledge on preventive practices, represented by the combination of direct and indirect effects (c1 + a1 × b1 + a2 × b2 + a3 × b3), demonstrates a Cohen’s f^2^ = 0.166, with a standard error of 0.057 and a value of z = 2.917. The statistical significance of this effect is *p* = 0.004, with a 95% confidence interval that ranges between 0.055 and 0.172. These results indicate that knowledge has a positive and significant overall association with preventive practices, with a standardized estimate of 0.278. According to Cohen’s guidelines, f^2^ = 0.166 indicates a medium effect size, suggesting a meaningful association between knowledge and preventive practices.

On the other hand, the total indirect effect, which represents the combined pathways through the variable’s occupation, school level and sex (a1 ∗ b1 + a2 ∗ b2 + a3 ∗ b3), presents a Cohen’s f^2^ = 0.027, with a standard error of 0.036 and a value of z = 0.764. The significance test indicates a *p* value of 0.445, and the 95% confidence interval varies between −0.043 and 0.097, including the zero value. This suggests that the indirect effects are not statistically significant, with a standardized estimate of 0.028 and f^2^ = 0.027 indicating a small effect size, which demonstrates a marginal contribution of the mediating variables to the total association between knowledge and preventive practices.

## 5. Discussion

The results of this study provide significant evidence of the influence of knowledge on preventive practices for multidrug-resistant tuberculosis, as well as the mediating role of sociodemographic variables in this relationship.

With respect to Hypothesis 1, which raised the direct influence of knowledge on preventive practices, the results revealed a positive and significant relationship (β = 0.194, *p* < 0.001) with a moderate total effect size (f^2^ = 0.166). This finding aligns with the broader literature demonstrating positive associations between disease knowledge and preventive behaviors, though our effect size appears more modest than some previous reports. For instance, [49] reported that treatment success is significantly related to disease understanding, while [50] documented that inadequate knowledge results in poor preventive practices. Our observed effect (β = 0.194) suggests a somewhat weaker knowledge-practice relationship compared to these earlier studies, potentially reflecting the unique challenges of MDR-TB compared to drug-susceptible tuberculosis. [51] identified inadequate knowledge as a significant barrier to preventive practice adherence, and [52] demonstrated how insufficient understanding increases transmission risk. The confirmation of this hypothesis, despite the moderate effect size, underscores that educational interventions remain essential for improving preventive practices in MDR-TB patients, though knowledge alone explains only a portion of behavioral variance.

Regarding Hypothesis 2, which proposed the mediation of occupation, the results did not show a significant indirect effect (β = −0.010, *p* = 0.490). This finding contrasts with several previous investigations documenting occupational influences on health behaviors. [2] reported that workers with more training exhibited better preventive practices, while [39,40] identified significant occupational influences on preventive measure application. The rejection of this hypothesis in our patient population may be explained by the high unemployment rate (23.28%) in our sample, which substantially differs from studies examining employed healthcare workers or general populations. This demographic characteristic may have limited our statistical power to detect occupational pathways, as the majority of participants lacked stable employment that would structure their daily routines and resource access.

Hypothesis 3, concerning the mediation of educational level, was confirmed with a significant indirect effect (β = 0.073, *p* < 0.001). This result is consistent with findings from [36], who reported that students with higher educational levels presented better preventive practices, and [38], who demonstrated educational level’s influence on tuberculosis-related attitudes and practices. However, our observed indirect effect (β = 0.073) is notably smaller than the direct effect of knowledge (β = 0.194), indicating that while educational level facilitates knowledge translation into preventive practices, it accounts for only a modest proportion of this relationship. This suggests that educational attainment enhances health literacy and information processing capacity, but additional unmeasured factors likely play substantial roles in behavioral implementation.

Finally, Hypothesis 4 on the mediation of sex was rejected (β = −0.035, *p* = 0.150). While [53] reported significant gender differences in preventive practices among high school students, our results align more closely with [43], who found no significant gender differences in knowledge and practices among medical students. The absence of sex-based mediation in our study requires careful interpretation within the broader empirical landscape. This convergence with more recent evidence may reflect several contextual factors specific to our study population and setting. First, Peru’s tuberculosis control program operates under a universal access framework that has progressively reduced gender-based barriers to healthcare services, potentially equalizing opportunities for health information acquisition and practice implementation across sexes. Second, the severity of MDR-TB diagnosis may serve as a powerful motivator that transcends traditional gender roles and social norms, compelling both male and female patients to prioritize preventive behaviors regardless of pre-existing gender-related health attitudes. Third, our sample consisted exclusively of patients already engaged in treatment, representing a self-selected group with demonstrated healthcare engagement that may not reflect broader population-level gender disparities. The mixed findings across studies suggest that sex operates as a context-dependent rather than universal mediator in health behavior adoption.

### 5.1. Theoretical and Practical Implications

The findings of this research contribute significantly to the theoretical understanding of preventive practice adoption in multidrug-resistant tuberculosis contexts. The proposed model extends existing knowledge by integrating sociodemographic factors as statistical mediators in the knowledge-practice relationship, providing a more comprehensive perspective of the mechanisms that may influence MDR-TB prevention. Particularly significant is the identification of educational level as a modest but consistent mediator, which enriches existing theoretical frameworks on preventive health behaviors. The empirical validation of these associations in the Latin American context broadens the knowledge base, which has traditionally focused on Western and Asian populations.

From a practical perspective, the results offer valuable guidelines for health institutions operating within Peru’s current tuberculosis control framework. Peru’s National Health Strategy for Tuberculosis Prevention and Control (ESNPCT) currently operates under a comprehensive model that includes diagnostic, treatment, and follow-up protocols, with 73% of MDR-TB patients treated through the Ministry of Health (MINSA) network [54,55]. The confirmation of the direct effect of knowledge and the mediating effect of educational level suggests the need for differentiated educational strategies that complement the existing ESNPCT framework according to patients’ educational backgrounds.

Educational interventions should be structured along multiple dimensions to accommodate diverse educational levels. For patients with limited formal education, interventions should prioritize simplified visual aids, including pictographic medication schedules, illustrated infection control demonstrations, and culturally appropriate audiovisual materials that minimize reliance on written text. Interactive group sessions using role-playing and practical demonstrations can reinforce key concepts without requiring advanced literacy. For patients with secondary or higher education, interventions can incorporate more detailed written materials explaining disease mechanisms, treatment protocols, and evidence-based preventive strategies. Personalized counseling sessions should be stratified by educational background, with healthcare providers trained to assess comprehension levels and adapt communication strategies accordingly. Technology-assisted interventions, including mobile health applications with literacy-appropriate interfaces, may enhance engagement across educational strata.

These educational approaches should be integrated into the three pillars of Peru’s current tuberculosis control strategy, particularly enhancing the patient-centered care component outlined in the WHO’s “End TB Strategy,” which Peru has adopted [55]. Although occupation and sex did not demonstrate significant mediating effects in our sample, these factors should not be dismissed in intervention design, considering Peru’s diverse population characteristics and the existing ESNPCT infrastructure. The current model emphasizes patient-centered care, evidence-based policies and support systems, and intensified research and innovation as outlined in the End TB Strategy framework.

Beyond educational and behavioral interventions, a broader preventive perspective is warranted. Recent evidence highlights the potential of vaccine-based strategies for mitigating antimicrobial resistance by reducing infection incidence and antibiotic dependence, suggesting that future MDR-TB prevention frameworks could integrate immunization research with educational and behavioral interventions to strengthen long-term disease control. This integrated approach, combining patient education, behavioral support, and emerging preventive technologies, may offer more comprehensive disease control than single-component interventions.

### 5.2. Limitations and Future Studies

This study has several limitations that must be considered when interpreting the results. The cross-sectional nature of the research precludes establishing causal relationships between variables; observed associations reflect correlational patterns that may result from multiple directional pathways or unmeasured confounding factors. Future research should employ longitudinal or experimental designs to test causal hypotheses, such as randomized controlled trials evaluating tailored educational interventions stratified by baseline educational level, or prospective cohort studies tracking how knowledge acquisition temporally precedes changes in preventive practices.

The use of self-reported questionnaires may have introduced recall bias and social desirability bias, potentially leading to overestimation of both knowledge and preventive practices. Participants may have reported idealized behaviors rather than actual practices, particularly given the interviewer-administered format. The potential for interviewer bias during face-to-face data collection could have influenced participant responses, especially regarding sensitive health behaviors. Interviewers, despite training, may have inadvertently conveyed expectations or judgments that shaped responses. Future studies should consider incorporating objective measures of preventive practices, such as medication adherence monitoring through electronic devices, observational assessments of infection control behaviors, or biomarkers indicating treatment compliance.

Unmeasured confounding variables, including access to healthcare services, household density, quality of housing ventilation, social support networks, transportation availability, and financial resources, were not controlled and may have influenced the observed associations. These structural and social determinants could simultaneously affect knowledge acquisition and preventive practice implementation, potentially confounding the relationships we examined. The exclusion of severely ill patients or non-Spanish speakers may have reduced representativeness in the broader MDR-TB population, limiting generalizability to these vulnerable subgroups who may face the greatest barriers to preventive behavior adoption.

The nonprobability convenience sampling, although justified by the characteristics of the specialized patient population, limits the generalizability of results to other patient populations or geographic contexts. Additionally, the research focused on health facilities in three specific districts in Peru, which may not fully reflect the realities of other regions with different cultural contexts, healthcare infrastructure, or tuberculosis burden.

For future research, expanding the geographical scope to include diverse regions and cultural contexts would allow a more complete understanding of how contextual factors influence preventive practice adoption. It would be valuable to examine additional mediating variables such as self-efficacy, health locus of control, perceived social support, and stigma, which could better explain the substantial unexplained variance (70.3%) in preventive practices observed in our model. Future research could benefit from incorporating mixed methods, complementing quantitative data with qualitative analyses that provide a deeper understanding of individual experiences of MDR-TB patients, barriers to behavioral change, and facilitators of successful preventive practice implementation.

## 6. Conclusions

This study provides empirical evidence on factors associated with preventive practices for multidrug-resistant tuberculosis among patients in Piura, Peru, contributing to the limited behavioral research in Latin American and low-resource settings where the global tuberculosis burden is concentrated. The findings demonstrate that knowledge is significantly associated with preventive practices, with educational level serving as the only significant mediator among the sociodemographic variables examined, while occupation and sex showed no significant mediating pathways. This context-specific evidence addresses a critical gap, as Peru ranks among the 30 high MDR-TB burden countries globally, yet patient-level behavioral research remains scarce. From a theoretical perspective, this study extends health behavior frameworks by documenting how educational level operates as a statistical mediator in the knowledge-practice relationship, suggesting that cognitive processing capacity and health literacy may constitute more salient pathways than occupational resources or gender-based social roles in this specific population and cultural context. The policy implications are substantial for tuberculosis control programs in high-burden settings: our findings demonstrate that literacy-appropriate educational materials and stratified patient counseling may strengthen preventive behavior adoption, yet the modest variance explained underscores that educational interventions alone have limited impact. Comprehensive prevention strategies must simultaneously address structural barriers, including healthcare access, socioeconomic resources, and household conditions that enable or constrain behavioral implementation. Peru’s National Health Strategy for Tuberculosis Prevention and Control, and similar programs across Latin America, should integrate multi-level interventions combining patient education with social support systems, economic assistance for treatment adherence, household-level infection control resources, and emerging preventive technologies such as vaccine-based approaches. Only through such comprehensive, evidence-based approaches grounded in local contexts can tuberculosis control programs meaningfully reduce MDR-TB transmission and improve treatment outcomes in the populations most affected by this disease.

## Figures and Tables

**Table 1:** Sociodemographic characteristics of the study participants (*n* = 280).

Variable	Category	*n*	%
Age			
Mean ± SD (range)	34.2 ± 12.8 years (18–74)	-	-
18–20 years	18–20	25	9.54
21–30 years	21–30	87	33.21
31–40 years	31–40	58	22.14
41–50 years	41–50	45	17.18
51–60 years	51–60	27	10.31
≥61 years	≥61	20	7.63
Sex			
Female	Female	128	48.85
Male	Male	134	51.15
Marital status			
Single	Single	58	22.14
Married	Married	48	18.32
Cohabiting	Cohabiting	57	21.75
Divorced	Divorced	46	17.56
Widowed	Widowed	53	20.23
Educational level			
No formal education	Illiterate	58	22.14
Primary education	Primary	56	21.37
Secondary education	Secondary	55	20.99
Technical education	Technical degree	42	16.03
University education	University degree	51	19.47
Place of residence			
Urban	Urban	101	38.55
Marginal urban	Marginal urban	84	32.06
Rural	Rural	77	29.39
Occupation			
Housewife	Housewife	52	19.85
Worker	Worker	55	20.99
Employee	Employee	50	19.08
Student	Student	44	16.79
Unemployed	Unemployed	61	23.28

**Table 2:** Model summary.

	Hayes Number ᵃ	AIC	AIC Weight	BIC	BIC Weight	Log-Likelihood	*n*	df
Mediation model	4	4080.153	1.000	4123.770	1.000	−2028.076	280	3

ᵃ Model configuration number defined by [48].

**Table 3:** R-squared.

	R^2^	R^2^ General
	Mediation Model	
Preventive_practices	0.297	0.117
Occupation	0.088	
school_level	0.056	
sex	0.028	

**Table 4:** Path coefficients.

								95% Confidence Interval		
			Label	*B*	Std. Error	z Value	*p*	Lower	Upper	Std. Estimate
Knowledge	→	Preventive_practices	c1	0.194	0.052	3.701	<0.001	0.091	0.265	0.200
Occupation	→	Preventive_practices	b1	0.023	0.033	0.697	0.486	−0.041	0.087	0.037
school_level	→	Preventive_practices	b2	0.308	0.050	6.168	<0.001	0.210	0.406	0.318
sex	→	Preventive_practices	b3	−0.402	0.049	−8.256	<0.001	−0.498	−0.307	−0.415
Knowledge	→	Occupation	a1	−0.462	0.089	−5.187	<0.001	−0.636	−0.287	−0.296
Knowledge	→	school_level	a2	0.236	0.058	4.069	<0.001	0.122	0.350	0.236
Knowledge	→	sex	a3	0.087	0.060	1.461	0.144	−0.030	0.204	0.087

Note: The arrows (→) indicate the relationship of the proposed hypothesis.

**Table 5:** Indirect effects.

										95% Confidence Interval		
					Label	*B*	Std. Error	z Value	*p*	Lower	Upper	Std. Estimate
Knowledge	→	Occupation	→	Preventive_practices	a1 × b1	−0.010	0.015	−0.690	0.490	−0.040	0.019	−0.011
Knowledge	→	school_level	→	Preventive_practices	a2 × b2	0.073	0.021	3.397	<0.001	0.031	0.115	0.075
Knowledge	→	sex	→	Preventive_practices	a3 × b3	−0.035	0.024	−1.439	0.150	−0.083	0.013	−0.036

Note: The arrows (→) indicate the relationship of the proposed hypothesis.

**Table 6:** Total effects.

									95% Confidence Interval		
				Label	f^2^	Std. Error	z Value	*p*	Lower	Upper	Std. Estimate
Total	Knowledge	→	Preventive_practices	c1 + a1 × b1 + a2 × b2 + a3 × b3	0.166	0.057	2.917	0.004	0.055	0.172	0.278
Total indirect	Knowledge	→	Preventive_practices	a1 × b1 + a2 × b2 + a3 × b3	0.027	0.036	0.764	0.445	−0.043	0.097	0.028

Note: The arrows (→) indicate the relationship of the proposed hypothesis.

## Data Availability

The data that support the findings of this study are available from the corresponding author upon reasonable request.
